# “Como conseguir a sua Doxi-PEP?”: vulnerabilidades e rotas críticas encontradas por homens no itinerário terapêutico no Brasil

**DOI:** 10.1590/0102-311XPT058524

**Published:** 2025-02-07

**Authors:** Álvaro Francisco Lopes de Sousa, Anderson Reis de Sousa

**Affiliations:** 1 Instituto de Ensino e Pesquisa, Hospital Sírio-Libanês, São Paulo, Brasil.; 2 Escola Nacional de Saúde Pública, Universidade NOVA de Lisboa, Lisboa, Portugal.; 3 Escola de Enfermagem, Universidade Federal da Bahia, Salvador, Brasil.

**Keywords:** Homens que Fazem Sexo com Homens, Profilaxia Pós-Exposição, Infecções Sexualmente Transmissíveis, Comportamento Sexual, Vulnerabilidade Sexual, Men Who Have Sex With Men, Post-Exposure Prophylaxis, Sexually Transmitted Diseases, Sexual Behavior, Sexual Vulnerability, Hombres que Hacen Sexo con Hombres, Profilaxis Posexposición, Enfermedades de Transmisión Sexual, Conducta Sexual, Vulnerabilidad Sexual

## Abstract

O objetivo desta pesquisa foi investigar o contexto de uso da profilaxia pós-exposição baseada em doxiciclina (Doxi-PEP) para a prevenção de infecções sexualmente transmissíveis (IST) por homens que fazem sexo com homens (HSH) no Brasil. Foi realizado um estudo qualitativo com 32 participantes HSH, selecionados por meio de redes sociais. A coleta de dados foi realizada utilizando questionários online, que incluíam perguntas abertas sobre experiências com a Doxi-PEP, acesso a medicamentos e interações com o sistema de saúde. As respostas foram analisadas usando o software IRaMuTeQ para classificação hierárquica descendente e análise temática reflexiva. Os resultados revelaram três categorias principais: (1) acesso e manejo da profilaxia pós-exposição, em que os participantes relataram dificuldades em obter a Doxi-PEP devido a restrições de prescrição e falta de informação; (2) percepções e conhecimentos sobre riscos e prevenção, que destacou uma mistura de conhecimento e desinformação sobre IST e estratégias de prevenção; (3) dinâmicas sociais e comportamentais, demonstrando como interações sociais e estigma influenciam práticas de prevenção. Este estudo também indicou a automedicação e o armazenamento de medicamentos como práticas comuns. Foram identificadas múltiplas barreiras no acesso e no manejo da Doxi-PEP entre HSH no Brasil, influenciadas por fatores individuais, sociais e programáticos. É imperativo desenvolver estratégias de saúde pública que melhorem o acesso e a informação sobre a Doxi-PEP entre os HSH, além de abordagens que reduzam o estigma associado às IST.

## Abstract (en)

This study aimed to investigate the context of doxycycline-based post-exposure prophylaxis (Doxy-PEP) use for the prevention of sexually transmitted infections (STIs) by men who have sex with men (MSM) in Brazil. A qualitative study was conducted with 32 MSM participants, selected on social media. Data was collected by online questionnaires, which included open-ended questions about experiences with Doxy-PEP, access to medications, and interactions with the health care system. Responses were analyzed using the IRaMuTeQ program for descending hierarchical classification and reflective thematic analysis. The results revealed three main categories: (1) access to and management of post-exposure prophylaxis, in which participants reported difficulties in obtaining Doxy-PEP due to prescribing restrictions and lack of information; (2) perceptions and knowledge on risks and prevention, which highlighted a mix of knowledge and misinformation about STIs and prevention strategies; (3) social and behavioral dynamics, demonstrating how social interactions and stigma influence prevention practices. The study also indicated self-medication and drug storage as common practices. The study identified multiple barriers to access and management of Doxy-PEP among MSM in Brazil, influenced by individual, social, and programmatic factors. It is imperative to develop public health strategies that improve access to and information about Doxy-PEP among MSM, as well as approaches that reduce STIs-associated stigma.

## Abstract (es)

El objetivo fue investigar el contexto de uso de Doxi-PEP (profilaxis posexposición a base de doxiciclina) para prevenir infecciones de transmisión sexual (ITS) por hombres que tienen sexo con hombres (HSH) en Brasil. Se realizó un estudio cualitativo con 32 participantes HSH, seleccionados por medio de redes sociales. La recopilación de datos se realizó mediante cuestionarios en línea, que incluían preguntas abiertas sobre experiencias con Doxi-PEP, acceso a medicamentos e interacciones con el sistema de salud. Las respuestas se analizaron usando el software IRaMuTeQ para clasificación jerárquica descendente y análisis temático reflexivo. Los resultados revelaron tres categorías principales: (1) acceso y manejo de la profilaxis posexposición, en que los participantes relataron dificultades para obtener Doxi-PEP debido a restricciones de prescripción y falta de información; (2) percepciones y conocimientos sobre riesgos y prevención, que destacó una mezcla de conocimiento y desinformación sobre las ITS y las estrategias de prevención; (3) dinámicas sociales y conductuales, que demuestran la manera en que las interacciones sociales y el estigma influyen en las prácticas de prevención. El estudio también indicó como prácticas comunes la automedicación y el almacenamiento de medicamentos. El estudio identificó múltiples barreras para el acceso y manejo de Doxi-PEP entre HSH en Brasil, influenciadas por factores individuales, sociales y programáticos. Es imperativo desarrollar estrategias de salud pública que mejoren el acceso y la información sobre Doxi-PEP entre HSH, así como enfoques que reduzcan el estigma asociado con las ITS.

## Introdução

A prevenção das infecções sexualmente transmissíveis (IST) representa um desafio persistente e complicado para a saúde pública global, diante das mudanças comportamentais, tecnológicas, de acesso às medidas de prevenção combinada, o que exige esforços contínuos para desenvolver e aprimorar estratégias eficazes. A profilaxia pré-exposição (PrEP) e a profilaxia pós-exposição (PEP) surgiram como intervenções promissoras, oferecendo uma linha adicional de proteção contra as IST para pessoas e grupos em situações de alto risco. No entanto, a eficácia dessas abordagens levanta questões complexas que se entrelaçam com itinerários terapêuticos individuais ^1^ e coletivos ^2^, especialmente em contextos marcados por vulnerabilidades significativas.

Especialmente no caso da PEP, a medida de prevenção tem caráter de urgência e deve ser utilizada em situação de risco para a infecção pelo HIV, por meio do uso de medicamentos ou imunobiológicos, os quais são capazes de reduzir o risco da aquisição de IST. Além disso, estratégias vêm sendo desenvolvidas com a finalidade de ampliar a cobertura de prevenção para além do HIV, a exemplo da sífilis, da clamídia e da gonorreia ^3^ ^,^ ^4^ ^,^ ^5^ ^,^ ^6^.

Um contexto já oficializado no cenário norte-americano é o uso da doxiciclina na prevenção de IST bacterianas após exposição sexual de risco, conhecida popularmente como Doxi-PEP (doxiciclina como profilaxia pós-exposição). A aprovação oficial dessa estratégia pelo órgão regulatório americano ^7^ abre uma discussão inevitável que parece estar sendo feita por usuários dos sistemas de saúde em vários países independentemente da disposição de órgãos oficiais e regulatórios ^8^: O uso da doxiciclina para a prevenção de IST pode ser feito *off-label* (fora da indicação da bula)?

É importante destacar que a implementação dessas estratégias nos Estados Unidos ocorre em um contexto marcado por uma regulação biofarmacológica fortemente influenciada por interesses mercadológicos. Conforme observado pelo sociólogo Nikolas Rose ^9^, essas tecnologias da vitalidade são manipuladas dentro de uma (bio)economia, com explícitos interesses comerciais na formação de consumidores de saúde. Isso se reflete na maneira como essas drogas são disponibilizadas e reguladas, muitas vezes como *off-label* ou *over-the-counter* (OTC), apontando para uma concepção neoliberal de saúde que privilegia a privatização da prevenção ^10^ ^,^ ^11^.

No Brasil, embora as profilaxias (PEP e PrEP) tenham sido incorporadas pelo Sistema Único de Saúde (SUS) na última década, promovendo mudanças positivas ^12^, é necessário considerar os desafios e as implicações dessa abordagem. O ponto crítico principal, nesse caso, reside na possibilidade de que tais terapias possam transferir responsabilidades coletivas de saúde para os indivíduos, especialmente para grupos historicamente estigmatizados. Além disso, a falta de políticas macroestruturais adequadas pode deixar essas pessoas desassistidas, obrigando-as a negociar essas estratégias de prevenção sozinhas.

Devido à rápida disponibilização de informações e à busca crescente por maneiras de autocuidado ^13^, especialmente entre populações vulnerabilizadas, é crucial considerar as evidências científicas de diversos ensaios clínicos ^3^ ^,^ ^4^ ^,^ ^5^ ^,^ ^6^ que demonstraram uma redução drástica nas taxas de incidência de gonorreia, clamídia e sífilis em populações-chave que utilizaram a doxiciclina, bem como disseminar essa informação em redes sociais e fóruns online. Nesse contexto, torna-se desafiador mensurar a potência e o efeito dessas informações em populações frequentemente afetadas por IST e seus efeitos no uso *off-label* da doxiciclina sem estudos específicos com as populações locais.

Diante desse contexto, compreender e abordar os itinerários terapêuticos percorridos pelos sujeitos, assim como as rotas críticas e as vulnerabilidades vivenciadas, é particularmente crucial para populações marginalizadas, como os homens que fazem sexo com homens (HSH). Esse grupo, frequentemente situado em um contexto de maior exposição ao HIV e outras IST devido a uma combinação de fatores sociais, econômicos, culturais, comportamentais, territoriais/geográficos, precisa ser considerado na agenda de prioridades em saúde pública em seus países, especialmente os mais afetados pela desigualdade social.

No Brasil e em todo o mundo, os HSH continuam a encontrar barreiras significativas no acesso a serviços de saúde apropriados e responsivos, adaptados às suas necessidades. A estigmatização, a discriminação e a falta de conhecimento específico entre os profissionais de saúde são apenas algumas das barreiras que podem impedir o acesso efetivo a estratégias de prevenção, como a PrEP e a PEP, destacando a importância dos itinerários terapêuticos na busca por soluções de cuidados ^14^ ^,^ ^15^.

Esses itinerários, ou os caminhos percorridos pelos indivíduos em busca de atendimento de saúde, são influenciados por uma multiplicidade de fatores, incluindo o acesso a informações confiáveis, a disponibilidade de recursos de saúde e as atitudes e práticas dentro das comunidades médicas e leigas ^1^ ^,^ ^2^. No caso dos HSH no Brasil, esses caminhos podem ser particularmente intrincados, navegando não apenas pelo sistema de saúde, mas também por um ambiente social que muitas vezes falha em reconhecer ou validar suas experiências e necessidades.

Portanto, o estudo dos itinerários terapêuticos adotados pelos HSH no Brasil e as vulnerabilidades enfrentadas por essa população no acesso à saúde representam um campo vital de investigação. A questão que guiou esse estudo foi: qual o contexto de uso, os itinerários terapêuticos e as vulnerabilidades enfrentados por HSH no Brasil para a obtenção e a utilização da Doxi-PEP para prevenir IST bacterianas? Este estudo tem o objetivo de investigar o contexto de uso da Doxi-PEP para a prevenção de IST por HSH no Brasil a fim de investigar o itinerário terapêutico adotado.

## Métodos

### Desenho do estudo e amostra

Este estudo utiliza uma abordagem qualitativa, investigando as experiências, as percepções e os itinerários terapêuticos de HSH no Brasil sobre o uso *off-label* da Doxi-PEP para a prevenção de IST bacterianas. A metodologia qualitativa foi empregada para permitir um entendimento profundo das experiências pessoais, dos desafios e das barreiras enfrentados pelos participantes.

O estudo se insere no projeto multicêntrico *In_PrEP* ^14^ ^,^ ^15^, que visa avaliar o acesso e a utilização da PrEP e PEP entre os HSH em todo o território brasileiro.

A população do estudo consistiu em homens adultos, os quais se identificavam com todos os aspectos convergentes ao seu “gênero de nascença” - orgão sexual, características físicas e papéis sociais atribuídos pela sociedade, reconhecendo-se enquanto homem, a partir da sua identidade de gênero - homens cisgêneros, que mantiveram relações sexuais com outros homens nos 12 meses anteriores a pesquisa, residentes do Brasil durante o período investigado. Foram excluídos do estudo os turistas no Brasil e cidadãos brasileiros residindo no exterior, para garantir imersão no contexto nacional das políticas de saúde em foco.

### Produção de dados

A produção dos dados envolveu a coleta, que foi realizada entre setembro e dezembro de 2022. Esse período permitiu capturar uma ampla gama de experiências e percepções sobre o uso da Doxi-PEP em diferentes estágios de discussão ao redor do mundo ^8^.

Inicialmente, participantes do *In_PrEP* que relataram o uso da Doxi-PEP foram identificados e triados. Para participar no projeto *In_PrEP*, um convite foi feito utilizando canais da internet, devido à natureza nacional e abrangente do projeto. Os participantes que concordaram em participar de uma subfase qualitativa do projeto foram direcionados para uma etapa qualitativa, online e detalhada, projetada para coletar informações profundas sobre suas experiências com a Doxi-PEP.

A pesquisa foi desenvolvida e administrada pelos pesquisadores, assegurando anonimato e confidencialidade para os participantes. As perguntas abordaram tópicos como o acesso à Doxi-PEP, experiências de uso, a percepção de riscos de IST, as interações com profissionais de saúde e a influência do uso da Doxi-PEP em seus comportamentos sexuais, permitindo que os participantes descrevessem suas experiências e opiniões de maneira detalhada. Os autores do estudo foram os responsáveis pela coleta de dados e entrevistas. Eles se identificam como gays e têm formação acadêmica e profissional em saúde pública, epidemiologia e ciências sociais, com experiências diversas em pesquisa qualitativa e quantitativa, que colocam em cena ante o objeto investigado, em termos de posicionamento crítico-reflexivo. Adotou-se uma postura empática, aberta e criativa na obtenção/produção dos dados, minimizando qualquer influência indevida sobre os participantes. No entanto, é importante destacar que a posição dos pesquisadores, por apresentar atravessamentos identitários e trajetórias com a problemática, pode ter influenciado a interação com os participantes e a interpretação dos dados.

### Formulário

Antes da implementação, o formulário (instrumento semiestruturado) foi rigorosamente validado - garantindo clareza, relevância e precisão da aparência/conteúdo. A revisão foi realizada por cinco especialistas em saúde sexual/prevenção de IST e cinco HSH da comunidade-chave, assegurando a sensibilidade cultural apropriada ao ambiente brasileiro. Essa etapa foi crucial para ajustes de linguagem, formato e fluxo de aplicação do instrumento alinhado às experiências/expectativas dos participantes.

Foram coletadas informações sociodemográficas e de saúde sobre: idade, identidade de gênero, orientação sexual, estado civil, raça/etnia, nível de educação e renda. Além disso, informações sobre a saúde sexual dos participantes, como o número de parceiros sexuais e práticas de sexo seguro, foram coletadas para fornecer um contexto melhor para as respostas. Em seguida, foram feitas quatro perguntas abertas com o objetivo de investigar detalhadamente as experiências dos participantes com a Doxi-PEP. Essas perguntas visavam compreender como os HSH tomaram conhecimento da Doxi-PEP, os processos e os desafios associados à sua obtenção, as práticas de administração e dosagem e as percepções sociais e pessoais relacionadas ao seu uso.

As respostas às perguntas abertas foram cruciais para revelar a complexidade das experiências dos HSH com a Doxi-PEP, permitindo que os participantes relatassem com suas próprias palavras como se engajam com essa estratégia de prevenção. Isso forneceu uma visão valiosa sobre as barreiras, os desafios e os facilitadores que influenciam a adoção e o uso contínuo da Doxi-PEP, bem como as atitudes e normas sociais em torno de seu uso dentro da comunidade HSH.

### Análise de dados

O processo de análise de dados empregou procedimentos rigorosos para assegurar a qualidade e a integridade das informações coletadas. As entrevistas foram transcritas e refinadas para aderir às normas gramaticais, concordâncias verbais, expressões idiomáticas e ao vocabulário da língua portuguesa. Todas as respostas dos participantes às perguntas qualitativas foram examinadas utilizando uma análise de dados abrangente.

As respostas obtidas das entrevistas foram submetidas a uma análise lexical usando o software IRaMuTeQ (*Interface de R pour les Analyses Multidimensionnelles de Textes et de Questionnaires, versão 7 alpha 2*, http://www.iramuteq.org/). A análise lexical, uma abordagem fundamental na pesquisa qualitativa, visa compreender o significado e a estrutura das palavras dentro de um texto. Essa técnica analítica foca na identificação de termos-chave, padrões linguísticos e relações semânticas encontradas no conteúdo textual, o que é essencial para organizar e interpretar informações em pesquisas qualitativas com grandes quantidades de texto.

Na fase inicial de nossa análise exploratória dos dados, realizamos a classificação hierárquica descendente (CHD) usando o IRaMuTeQ. Essa técnica, empregada em análises multivariadas, agrupa termos em diferentes níveis hierárquicos com base em suas semelhanças, formando um dendrograma. Seguindo uma lógica de descida, agrupa os termos mais comparáveis em classes maiores,. Essa metodologia forneceu uma visão estruturada das relações entre os elementos, permitindo uma compreensão visual das semelhanças e diferenças dentro do conjunto de dados, facilitando a análise temática.

Na segunda fase, uma análise de conteúdo reflexiva foi conduzida com base na CHD gerada pelo IRaMuTeQ ^16^. Após a revisão das classes semânticas formadas, *clusters* temáticos ou áreas específicas de interesse foram identificados, direcionando a análise de conteúdo para explorar mais a fundo o significado e o contexto desses agrupamentos. Entender as relações hierárquicas entre os elementos permitiu uma abordagem mais sofisticada e contextualizada na interpretação do conteúdo, fornecendo uma base sólida para a análise de conteúdo temático reflexiva ^16^.

Em seguida, empregamos essa análise, desenvolvida por Braun & Clarke ^17^. Dado o volume de dados e o desejo por reflexividade, adotamos uma abordagem de codificação fluida e flexível para alcançar a imersão e o engajamento profundo com os dados, justificando o processamento dos dados por meio do software. Assim, a análise envolveu uma interação contínua entre o banco de dados, a codificação de trechos textuais, a análise desses trechos, a identificação de padrões nos dados e sua comunicação, tudo visando revelar temas. Essa abordagem visa identificar, analisar e relatar padrões temáticos dentro dos dados, visando alcançar a reflexividade característica da pesquisa qualitativa. Os achados centrais foram interpretados à luz do marco teórico-conceitual de itinerários terapêuticos ^18^ e das dimensões da vulnerabilidade: individual, social e programática/institucional, conceituada por Ayres ^19^, e do conceito teórico-político de interseccionalidade, proposto por Crenshaw ^20^.

Além disso, incorporamos o conceito de interseccionalidade para complementar nossa análise de vulnerabilidade. A interseccionalidade nos permitiu considerar de que forma os múltiplos e simultâneos marcadores de identidade, como raça, gênero, escolaridade, renda e local de residência, interagem para potencializar as vulnerabilidades enfrentadas pelos HSH.

### Considerações éticas

O projeto foi aprovado pelo Comitê de Ética em Pesquisa da Escola de Enfermagem de Ribeirão Preto da Universidade de São Paulo (parecer nº 4.163.084), de acordo com os critérios estabelecidos pelas *Resoluções nº 466/2012*, *510/2016* e *674/2022* do Conselho Nacional de Saúde. Critérios de segurança e proteção de dados foram estabelecidos em conformidade com essas resoluções. O Termo de Consentimento Livre e Esclarecido foi assinado por todos os participantes, garantindo sua compreensão e concordância com os procedimentos de pesquisa. Para a garantia do anonimato, foi adotado um código: H, de homem, e o número de participação no estudo.

## Resultados

Participaram deste estudo 32 HSH, cisgêneros (100%), sendo: 18 (56,2%) da Região Sudeste do Brasil, seis (18,8%) da Sul, cinco (15,6%) da Centro-oeste e três (9,4%) do Nordeste. Todos (100%) residentes em áreas urbanas - zona metropolitana. Houve predominância de adultos com 26 anos ou mais (53,1%), solteiros (71,9%) e pardos (59,3%). Ademais, a vasta maioria possuía educação superior (84,4%), acesso à internet e uso de redes sociais digitais (100%). Eram economicamente ativos (100%), com empregabilidade formal (100%), salário/renda acima de R$ 5 mil (62,5%) e relataram ter mais de dois parceiros sexuais nos últimos 30 dias (84,4%), além de fazer uso de aplicativos para encontros afetivo-sexuais (84,3). Todos eram usuários de PrEP (100%).

**Figura 1:**
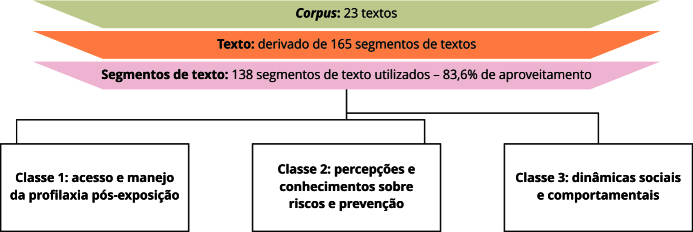
Dendrograma representando a classificação hierárquica descendente de palavras organizadas por classes. Brasil, 2022.

A análise se baseou em 32 respostas abertas que, após serem processadas pelo programa IRaMuTeQ, resultaram em um *corpus* de 165 segmentos de texto. Desses, 138 segmentos, ou 83,6%, foram identificados pelo software como relevantes para análises posteriores. O processamento objetivou extrair indicadores significativos das respostas dos entrevistados. Por meio da CHD, foram identificadas três principais classes temáticas, representando conjuntos de expressões e palavras utilizadas pelos participantes para transmitir significados relacionados aos tópicos discutidos. As classes temáticas identificadas foram descritas conforme visualizado na Figura 1.

Cada categoria reflete um conjunto específico de preocupações, comportamentos e interações relacionados Doxi-PEP e ao gerenciamento da saúde sexual (Figura 1).

### Classe 1: acesso e manejo da profilaxia pós-exposição

Esta categoria engloba os depoimentos que discutem o acesso à doxiciclina e a outros antibióticos utilizados como profiláticos, bem como as estratégias para obtê-los por meio de receitas e o manejo pessoal da medicação adotadas pelos homens pesquisados:

“...*há alguns dias eu li alguns artigos que falavam de utilizar uma dose única de 200mg em até 72h após ter uma relação sexual desprotegida e pensei em aderir. No entanto, a falta de informação, dificulta a situação* (...) *por exemplo, eu não encontrei nenhuma informação que entrasse em um consenso se a prescrição deve mesmo ser essa quantidade a ser tomada ou não, até porque os artigos estão em inglês*” (H-12).

“...*eu faço uso de PrEP* [HIV] *desde que ficou disponível no Brasil em 2018, se tem um tratamento em potencial para outra IST, porque não utilizar? Sinceramente, é encorajador saber que existe uma possibilidade de tratar outras IST, ainda mais que já tem pesquisas indicando o uso da doxiciclina contra a sífilis e a clamídia*” (H-7).

Os principais pontos dessa classe dizem respeito ao “acesso restrito a esses medicamentos”, à medida que os indivíduos enfrentam dificuldades para obter as receitas e adquirir os medicamentos devido a questões como as regulamentações e as restrições com a prescrição, bem como aos meios que utilizam para conseguir os medicamentos.

Nessa classe, destacam-se ainda, os relatos de “manipulação de informações” e o uso de redes sociais digitais para obter as receitas ou informações acerca da aquisição dos medicamentos, conforme demonstrado a seguir:

“...*não é fácil conseguir a doxiciclina, precisa de receita. Não é igual a ritalina que você compra direto de traficante* (...) *uma amiga que me prescreveu um caixa, mas confesso que não estou usando do jeito que ela indicou, e sim do jeito que está na internet e nos artigos que li* (H-15).

“...*é fácil para conseguir a receita, ou buscar por uma farmácia de bairro que vende essas coisas sem receita. E assim, esse assunto foi divulgado e ficou comum falar sobre isso com os amigos. Então, eu me arrisquei* (...) *tive sífilis e a médica me receitou benzetacil aplicado, claro. Mas não foi a minha primeira vez, e menti para ela que tinha reação ao benzetacil e então pedi para que me prescrevesse a doxiciclina* (...). *Isso foi sugestão de um amigo que havia passado por situação parecida com a minha, mas depois da doxiciclina, ficou igual a mim, sem a “dor infernal” que o benzetacil causa* (...) *quando a médica me passou a doxi eu interrompi meu tratamento na metade, quando acabou os sintomas e guardei os comprimidos que sobrou. Aí, quando tenho alguma exposição mais arriscada eu tomo os meus comprimidos certinho, 24 horas depois da exposição*... (H-9).

“...*eu vi ensinando como usar a doxi no grupo de Facebook e deu tudo certo: você diz ao médico que o seu parceiro foi diagnosticado com sífilis secundária e que o médico dele pediu pra você ir atrás de se tratar da sífilis também, porque vocês transam ‘sem capa’. Aí o médico vai pedir o teste rápido, nesse caso você fala que já teve sífilis pra ele desistir e pedir o VDRL que só vai sair em sete dias. Assim, ele vai ter que seguir o protocolo e te passar um monte de benzetacil, então, você fala que tem reação e ele te passa duas caixas de doxiciclina, de boa* (...) *se você não for uma pessoa muito transante, o medicamento dura pra um bom tempo, né?* (...) *depois de usar a doxi eu não mais tive nada, nenhuma IST. Antes eram três aplicações de benzetacil por ano, quase, e assim, todas as doses na bunda* [intramuscular]” (H-22).

O autogerenciamento de doses e medicações, por meio de práticas de automedicação e interrupção do tratamento para armazenamento de medicamentos para uso futuro, é um importante destaque para a garantia de medicação futura e a gestão de prevenção de IST futuras:

“...*tive sífilis, a médica quis me enfiar a benzetacil, mas eu já tinha passado por isso antes e pedi a doxiciclina. O tratamento foi mais tranquilo, sem a ‘dor insana’ do benzetacil que tomei. O que faço agora é guardar os comprimidos para usar em situações com ‘risco’, por exemplo surubas ou darkroom*” (H-13).

“...*ela* [a médica] *nunca indicou, mas eu já interrompi tratamento para guardar doxi. Aí, é pegar na farmácia e seguir o plano*” (H-27).

### Classe 2: percepções e conhecimentos sobre riscos e prevenção

Esta categoria abrange depoimentos que refletem sobre a exposição a IST, a percepção e o medo da resistência bacteriana e as estratégias de prevenção adotadas. Os principais pontos são a dualidade entre o desejo de combinar estratégias como Doxi-PEP e PrEP, ao mesmo tempo em que existe uma preocupação com o risco de resistência bacteriana e outros problemas associados ao uso inadequado de antibióticos:

“...*eu fiz o uso da Doxi-PEP depois de uma suruba* [sexo em grupo] *na sauna, um parceiro lá me disse: tome esses dois comprimidos amanhã junto com os seus comprimidos* [dolutegravir e lamivudina], *aí fui lá e tomei. Ele ainda falou: ‘olha tem que ser no máximo 24 horas depois da exposição’. Ele me explicou que vive com HIV também e que tem sido indicado, para algumas IST* (...) *acho que é isso*” (H-10).

É notório o desejo dos participantes de encontrar métodos de prevenção para além da proteção ao HIV, abordando outras IST e considerando a Doxi-PEP como uma opção:

“...*eu não uso a PrEP, por só curtir sexo oral, mas isso me assusta às vezes, porque os meus amigos ficam enchendo meu saco sobre isso, falando pra eu utilizar. Mas aí, eu tava querendo alternativas e umas ideias sobre prevenção combinada pro sexo oral, porque já peguei gonorreia*” (H-16).

Essa categoria, no entanto, contempla dúvidas e incertezas também sob a eficácia do uso da Doxi-PEP a IST:

“(...) *eu tomava essa dose duas horas antes da exposição e mais uma de 100mg 24 e 36 horas depois, parecido com a PrEP sob demanda, e até o momento deu certo* [não sei se é sorte ou eficiência da profilaxia]” (H-24).

“...*até agora, parece que tem dado certo, mas, claro, sempre rola aquela dúvida se é sorte ou a doxiciclina tá fazendo o efeito direito*” (H-11).

### Classe 3: dinâmicas sociais e comportamentais

Esta categoria abrange depoimentos que discutem o impacto das interações sociais, pressões de grupo e normas comportamentais na tomada de decisões relacionadas à saúde sexual.

Os principais pontos-chave são: o reconhecimento da influência das redes sociais e dos pares, conforme discussões sobre como redes de pares e a partilha de informações em grupos sociais influenciam as práticas de saúde são comumente vistas nos discursos. Reflexões sobre como o estigma associado às IST e à sexualidade pode impactar as escolhas de prevenção e tratamento; relatos que enfatizam a interação entre prazer, saúde e bem-estar, e como os indivíduos equilibram esses fatores em suas práticas sexuais.

“...*ultimamente, a minha experiência tem sido com a doxiciclina mesmo, principalmente quando rola uma exposição mais prolongada*” (H-08).

“...*quando falamos em risco sem considerar o monogâmico por aí que mal sabe usar a camisinha, quem dirá conhecer outros métodos de prevenção e daí não fazem exames regulares, mas vivem traindo, pagando de moralistas, a doxiciclina é muito útil para a autoproteção*” (H-32).

“...*eu uso a doxiciclina quando faço sexo em festa ou suruba, essas coisas que envolvem muita carga viral, sabe? Tipo em Madri, fui em uma festa e aprendi que se você não se cuida, o que acontece na festa não fica na festa*” (H-14).

**Figura 2:**
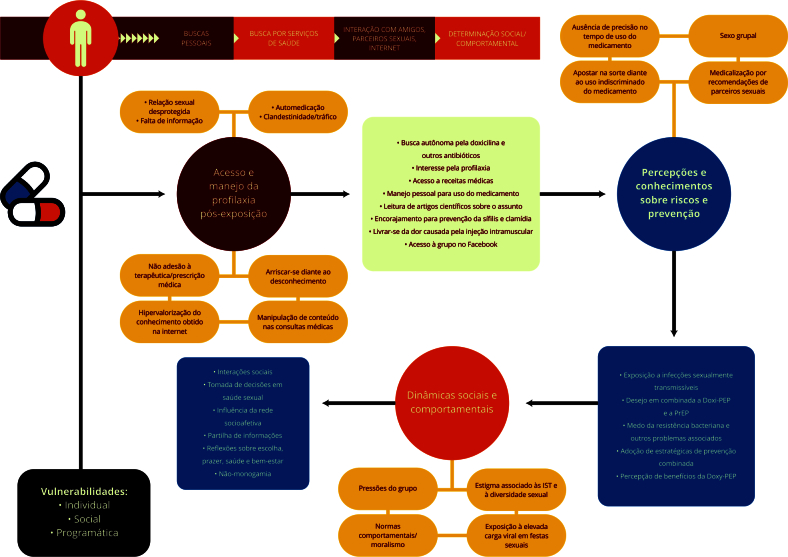
Itinerários terapêuticos de homens brasileiros em busca de Doxi-PEP. Brasil, 2022.

Doxi-PEP: doxiciclina como profilaxia pós-exposição; HSM: homens que fazem sexo com homens; PrEP: profilaxia pré-exposição.

Ao analisar os depoimentos sob a perspectiva do quadro de vulnerabilidade, podemos classificar os principais achados em diferentes dimensões da vulnerabilidade: individual, social e programática/institucional (Quadro 1 e Figura 2).

**Quadro 1:** Exercício teórico-reflexivo de enquadramento dos achados ao referencial teórico de vulnerabilidades sociais em saúde e de interseccionalidade. Brasil, 2022.

ENQUADRAMENTO TEÓRICO DOS ACHADOS ÀS DIFERENTES DIMENSÕES DA VULNERABILIDADE SOCIAL EM SAÚDE E INTERSECCIONALIDADE	
Vulnerabilidade individual	1. Conhecimento e informação: há uma lacuna crítica no conhecimento e compreensão sobre o uso correto da profilaxia pós-exposição. A confusão em torno das dosagens, regimes de tratamento e preocupações relativas à resistência bacteriana destacam uma necessidade urgente de educação e orientação específica para o usuário.
	2. Comportamento e práticas de saúde: existem pessoas que estão procurando ativamente métodos de prevenção, como a doxiciclina. No entanto, elas estão recorrendo à automedicação e interrompendo tratamentos, o que reflete um conflito entre o medo da infecção e os efeitos adversos. Isso demonstra um dilema, em que na busca por cuidar de si mesmos, incorrem na possibilidade de gerar danos.
	3. Consciência de prevenção: reconhece-se a importância da prevenção de ISTs, levando em consideração diversas práticas e comportamentos. Muitos buscam maneiras próprias de gerenciar essas precauções ou integrá-las em seu cotidiano, criando mecanismos para propiciar satisfação pessoal, saúde e bem-estar.
Interseccionalidade	O nível elevado de escolaridade apresenta-se em expressividade. Contudo, pode não implicar em bom grau de Letramento em Saúde sexual, no uso de medicamentos profiláticos e no automanejo da saúde-doença; o bom nível socioeconômico pode oportunizar o acesso os HSH pesquisados à rede de medicamentos, mas segue atravessado de comportamentos de saúde propenso a risco, o qual pode ser decorrente da desinformação, cultura midiática, apelo da indústria farmacêutica, clandestinidade, traços de personalidade; o aprendizado acerca da prevenção pode ter influência do perfil socioeconômico, localização geográfica/território e incorporação tecnológica ao cotidiano, mas pode ofuscar a desorientação e a autogestão eficaz do controle e da manutenção da saúde sexual.
Vulnerabilidade social	1. Estigma e normas sociais: o estigma associado às ISTs e à sexualidade, particularmente dentro da própria comunidade LGBT+, pode ter um impacto prejudicial nas práticas de prevenção e tratamento. Isso pode levar a abordagens discretas ou não convencionais na tentativa de evitar julgamento ou discriminação, destacando a necessidade de ambientes mais acolhedores e inclusivos.
	2. Acesso e disponibilidade: existe uma lacuna significativa entre a identificação de novos tratamentos e sua efetiva incorporação às políticas públicas de saúde. Devido a essa defasagem, as pessoas buscam acesso antecipado a medicamentos por conta própria. Desde 2020, a doxiciclina vem sendo relatada nos depoimentos, como usada na profilaxia pós-exposição de forma independente. Mas, em quase quatro anos, pouco foi feito para incorporar essa abordagem às estratégias de saúde pública do Brasil. A falta de acesso à doxiciclina, causada por restrições na prescrição ou barreiras sociais, destaca a necessidade de tornar medicamentos e serviços de saúde mais acessíveis e entendidos, especialmente para populações vulneráveis.
	3. Mídias sociais e comunicação: o papel das redes de pares na disseminação de informações, sejam elas precisas ou não, sobre a profilaxia pós-exposição destaca a influência significativa do contexto social nas decisões relacionadas à saúde, sublinhando a necessidade de estratégias eficazes de comunicação comunitária.
Interseccionalidade	A estigamtização e normatização social estrutural provoca um “angarrajamento” de fenômenos que se entrecruzam e produzem repercussões deletérias para a autopercepção de saúde, adoção de comportamentos seguros e conscientes, mediante ao suporte da rede de apoio institucional de saúde, que ainda se mostra ausente, punitiva e discriminatória para com as demandas apresentadas pelos usuários, o que faz das redes sociais digitais o espaço de compartilhamento das experiências entre os pares, criando novos caminhos e itinerários terapêuticos, os quais podem ser permeados por rotas críticas e iniquidades em saúde, especialmente, quando marcada pelo quesito raça/cor/etnia, classe social, idade/geração, religiosidade e gênero/sexualidade.
Vulnerabilidade programática/institucional	1. Políticas de saúde e práticas institucionais: a falta de diretrizes oficiais claras e protocolos específicos quanto ao uso da doxiciclina como PEP destaca uma lacuna nos programas de saúde pública, possibilitando práticas inconsistentes e potencialmente perigosas, e assim ressaltando a necessidade de diretrizes políticas bem definidas e baseadas em evidências.
	2. Resistência bacteriana: a preocupação com o desenvolvimento de resistência bacteriana sublinha a necessidade de abordagens mais sustentáveis e cientificamente informadas ao uso de antibióticos, bem como a importância do monitoramento contínuo e da pesquisa para orientar práticas seguras e eficazes.
	3. Relacionamento com profissionais de saúde: a variabilidade nas interações com profissionais de saúde, que variam de diálogos francos a estratégias de manipulação para obter prescrições, reflete as complexidades na relação paciente-fornecedor. Esta situação enfatiza a necessidade de comunicação clara, ética profissional e orientação explícita, fomentando uma relação de confiança e abertura entre pacientes e profissionais de saúde.
Interseccionalidade	Os sistemas de crenças e valores, atrelados aos marcadores sociais dos profissionais de saúde, bem como refletidos nas diretrizes das políticas públicas de saúde instituídas no Brasil, podem incidir no modo com a produção do cuidado, a assistência à saúde e a gestão dos serviços e a implementação das políticas públicas vão dar conta ou não de atender as demandas, necessidades e especificidades singulares dos HSH no contexto do emprego da Doxi-PEP, sob uma perspectiva interseccional que faça parte do cotidiano das práticas profissionais e dos serviços de saúde disponíveis na rede.

HSM: homens que fazem sexo com homens; IST: infecções sexualmente transmissíveis; PEP: profilaxia pós-exposição.

## Discussão

A análise detalhada dos dados obtidos de uma amostra de HSH no Brasil revela dinâmicas complexas no gerenciamento da PEP de IST bacterianas. Para o nosso melhor conhecimento, esta é a primeira pesquisa conduzida na América Latina a explorar essa realidade, evidenciando a singularidade e a contribuição inovadora de nosso estudo para o campo. Esta pesquisa apresenta um esforço por parte dos HSH em aprimorar a proteção em relação à sua saúde sexual, uma iniciativa que, à primeira vista, pode ser interpretada como proativa e responsável.

Uma análise mais detalhada do perfil social, demográfico e territorial/espacial dos participantes apontam para a manifestação interseccional do entrecruzamento de categorias/marcadores sociais da diferença ^20^, uma vez que que houve concentração de HSH das regiões Sudeste (56,2%) e Sul (18,8%) do Brasil, áreas conhecidas por serem economicamente mais desenvolvidas. Além disso, a vasta maioria possuía Ensino Superior (84,4%) e renda formal (75%), o que pode indicar um alto nível socioeconômico. Todos os participantes eram usuários de PrEP, mostrando que já têm acesso a estratégias de prevenção diferenciadas e que são proativos no cuidado com sua saúde sexual. No entanto, esse perfil pode não representar a realidade mais ampla dos HSH no Brasil, especialmente aqueles de regiões menos favorecidas economicamente, como o Norte e Nordeste, em que o acesso a recursos de saúde, como a PrEP, é mais limitado. Esse “viés da amostra” sugere que o debate e o uso mais imediato ou *off-label* da Doxi-PEP no Brasil, pode espelhar uma interseccionalidade de diferentes marcadores de identidade, como raça, gênero e classe social ^20^, bem como expressar um conjunto de vulnerabilidades individuais, sociais e programáticas ^21^.

Nos depoimentos, o engajamento na prevenção da saúde sexual é marcado por uma série de desafios significativos. Inicialmente, o uso *off-label* da doxiciclina sugere um cenário em que decisões de saúde são tomadas com base em informações incorretas, incompletas ou em entendimentos distorcidos sobre a medicação e seu uso seguro e eficaz ^20^ ^,^ ^21^. Uma das principais complicações, nesse caso, é a falta de orientação clínica formal e a ausência de regulamentações específicas que endossaram, no Brasil, o uso da doxiciclina como uma estratégia de prevenção legítima para IST nesse cenário ^22^ ^,^ ^23^ ^,^ ^24^. Portanto, mesmo que a intenção subjacente seja aprimorar a proteção contra IST, a base de conhecimento sobre a qual essas decisões são tomadas é frequentemente frágil e insuficientemente fundamentada, necessitando do suporte de profissionais de saúde.

A desinformação e o uso de informações incompletas criam um terreno fértil e amplo para práticas de saúde potencialmente prejudiciais ^25^ ^,^ ^26^. Tais práticas podem ser mitigadas ou agravadas em face do contexto social. Nesse aspecto, cabe reconhecer que a forma das desigualdades varia conforme a experiência, e o estigma também varia situacionalmente. Autores têm defendido a categoria analítica “estigma interseccional” como um meio mais compreensivo para o exame das vulnerabilidades, as quais interagem simultaneamente com estigmas prévios baseados raça/cor, etnia, classe social, origem geográfica, gênero, geração e sexualidade, por exemplo ^26^ ^,^ ^27^ ^,^ ^28^.

Sem um entendimento claro das dosagens corretas, dos regimes de tratamento ideais e das interações medicamentosas potenciais, articulados com a compreensão ampliada da interação ou da sobreposição de fatores sociais definidores da identidade e comportamento dos HSH, em termos dos impactos na sua relação com a sociedade e o acesso aos direitos ^19^, os indivíduos estão sujeitos a cometer erros que não apenas podem anular a eficácia pretendida da profilaxia, mas também expor esses indivíduos a riscos de saúde adicionais.

Por exemplo, a automedicação com doxiciclina sem supervisão médica adequada pode resultar em uso incorreto ou impreciso do medicamento, aumentando o risco de desenvolvimento de resistência bacteriana ^25^ ^,^ ^28^, que é um problema crescente e preocupante no tratamento de IST.

Além disso, essa abordagem *off-label*, ou não regulamentada, reflete uma lacuna mais ampla do próprio sistema de saúde que falha em fornecer informações acessíveis, compreensíveis e baseadas em evidências para HSH, um grupo historicamente marginalizado por outros fatores socioculturais e econômicos ^13^. A falta de comunicação clara e diretrizes específicas por parte dos profissionais de saúde contribui para que esses homens recorram a métodos alternativos de prevenção, orientados mais por boatos ou informações parciais obtidas por meio de redes sociais e comunidades online do que por conselhos médicos confiáveis.

Esse cenário não apenas representa uma ameaça à saúde individual dos envolvidos, mas também tem implicações mais amplas para a saúde pública. O envolvimento em práticas preventivas baseadas em desinformação pode contribuir para um aumento na prevalência de IST, minando os esforços de saúde pública para controlar e prevenir essas infecções ^6^ ^,^ ^25^ ^,^ ^28^.

Portanto, embora o engajamento desses indivíduos na prevenção de sua saúde sexual possa parecer um passo positivo, a realidade é que ele é construído sobre uma base instável de desinformação e práticas inadequadas. Isso claramente sublinha a importância do letramento em saúde e reforça a necessidade crítica de campanhas educativas direcionadas, do desenvolvimento de políticas de saúde pública inclusivas e abrangentes e da implementação de programas de prevenção que sejam acessíveis, culturalmente sensíveis e baseados em evidências para esse grupo. Somente dessa maneira podemos garantir que o esforço para aprimorar a prevenção da saúde sexual entre HSH seja eficaz e seguro, contribuindo positivamente tanto para a saúde individual quanto coletiva.

Dessa forma, este estudo desvenda um panorama multifacetado de comportamentos, conhecimentos e acesso a recursos de saúde sexual entre esse segmento populacional, ilustrando as várias dimensões de vulnerabilidades que afetam esse grupo.

Particularmente a vulnerabilidade social é exacerbada pelo estigma associado às IST e à sexualidade, dificultando práticas preventivas e de tratamento adequadas. Essa situação é evidenciada pela dificuldade de acesso e discussão com profissionais sobre a doxiciclina, reflexo da barreira imposta tanto por preconceitos quanto por limitações no sistema de saúde ^6^. Ao mesmo tempo, a influência das redes sociais e dos pares na disseminação de informações destaca a influência decisiva do contexto social nas decisões de saúde ^6^.

Quanto aos aspectos programáticos da vulnerabilidade, é notável a existência de uma lacuna significativa devido à falta de diretrizes explícitas e protocolos dedicados para a aplicação da doxiciclina como medida de PEP. Embora os achados da nossa pesquisa apresentem um cenário de 2022, percebeu-se que pouco progresso foi feito em termos de avanços nessa área de discussão até o momento, em 2024. Essa carência indica uma falha grave nas políticas de saúde, contradizendo os pilares do SUS no Brasil, que visa assegurar a universalidade, integralidade e equidade no acesso aos serviços de saúde. A ausência de diretrizes institucionais bem definidas sobre essa questão permite o uso não regulamentado de um medicamento cuja eficácia e segurança para esse propósito específico não foram adequadamente investigadas, representando riscos tanto para a saúde pública quanto individual.

### Limitações

Embora o método adotado permita uma exploração aprofundada das experiências e percepções individuais, a generalização dos resultados para a população mais ampla de HSH é limitada neste estudo. Além disso, a amostra foi selecionada de um subgrupo específico que já estava utilizando PrEP, o que pode reduzir a diversidade de experiências e comportamentos de saúde sexual encontrados. Isso sugere que os achados podem ser mais aplicáveis a indivíduos com um certo nível de envolvimento com os serviços de saúde e uma predisposição para buscar ativamente métodos de prevenção. Além disso, a utilização da plataforma IRaMuTeQ para análise de dados, embora potente, depende da interpretação dos termos e expressões utilizados pelos participantes.

## Considerações finais

A análise dos itinerários terapêuticos e as vulnerabilidades enfrentadas por HSH no Brasil para obter e usar doxiciclina como PEP para prevenir IST bacterianas revela um quadro complexo de desafios e adaptações. Na busca por proteção, esses homens enfrentam um cenário caracterizado por barreiras de acesso, lacunas informativas e dinâmicas sociais e comportamentais que influenciam fortemente suas práticas de saúde.

O contexto de uso da Doxi-PEP entre HSH no Brasil é caracterizado por um itinerário terapêutico que frequentemente começa e se desenvolve fora dos ambientes clínicos tradicionais. As mídias sociais e comunidades online se tornam, nesse contexto, espaços vitais para a troca de informações e experiências, nos quais HSH busca e compartilha conhecimentos sobre a aquisição e uso *off-label* da doxiciclina. Essa dinâmica destaca a resiliência desse grupo na busca por soluções para a proteção da saúde sexual na ausência de diretrizes claras e apoio institucional.

As vulnerabilidades enfrentadas são multifacetadas, abrangendo aspectos individuais, sociais e programáticos/institucionais. Individualmente, a falta de informações específicas e confiáveis sobre a Doxi-PEP leva a práticas de automedicação e uso não regulamentado do medicamento, aumentando assim o risco de efeitos adversos. Socialmente, o estigma associado às IST e à sexualidade influencia negativamente as decisões de saúde, reforçando barreiras para acessar cuidados apropriados e promovendo práticas preventivas clandestinas. Do ponto de vista programático, a ausência de políticas de saúde que reconheçam e abordem especificamente as necessidades dos HSH perpetua essas vulnerabilidades, limitando assim o acesso a intervenções preventivas como a Doxi-PEP.
